# Automated measurement of fast mitochondrial transport in neurons

**DOI:** 10.3389/fncel.2015.00435

**Published:** 2015-11-03

**Authors:** Kyle E. Miller, Xin-An Liu, Sathyanarayanan V. Puthanveettil

**Affiliations:** ^1^Department of Integrative Biology, Michigan State UniversityEast Lansing, MI, USA; ^2^Department of Neuroscience, The Scripps Research Institute, Scripps FloridaJupiter, FL, USA

**Keywords:** mitochondria, fast axonal transport, motion tracking, drug screening and neurons

## Abstract

There is growing recognition that fast mitochondrial transport in neurons is disrupted in multiple neurological diseases and psychiatric disorders. However, a major constraint in identifying novel therapeutics based on mitochondrial transport is that the large-scale analysis of fast transport is time consuming. Here we describe methodologies for the automated analysis of fast mitochondrial transport from data acquired using a robotic microscope. We focused on addressing questions of measurement precision, speed, reliably, workflow ease, statistical processing, and presentation. We used optical flow and particle tracking algorithms, implemented in ImageJ, to measure mitochondrial movement in primary cultured cortical and hippocampal neurons. With it, we are able to generate complete descriptions of movement profiles in an automated fashion of hundreds of thousands of mitochondria with a processing time of approximately one hour. We describe the calibration of the parameters of the tracking algorithms and demonstrate that they are capable of measuring the fast transport of a single mitochondrion. We then show that the methods are capable of reliably measuring the inhibition of fast mitochondria transport induced by the disruption of microtubules with the drug nocodazole in both hippocampal and cortical neurons. This work lays the foundation for future large-scale screens designed to identify compounds that modulate mitochondrial motility.

## Introduction

Considering the abnormalities in mitochondrial transport observed in neurological disease (De Vos et al., 2008), novel compounds that manipulate transport could have therapeutic value (Kadakkuzha et al., 2014). Development of high-throughput screening assays for mitochondrial transport is potentially an effective means for the discovery of such compounds (Carnero, 2006). With the development of automated microscopes (Mei et al., 2012) and the availability of libraries of small molecules (Kadakkuzha et al., 2014) it is now possible to generate in a single day raw datasets where the effects of hundreds of compounds can be analyzed. Although the analysis of mitochondrial transport in tens to hundreds of neurons is straightforward using manual tracking and kymographs (Miller and Sheetz, 2004), methodologies have not been developed for the large-scale analysis of mitochondrial transport data generated by robotic microscopes. With the long-term goal of screening large libraries of compounds, our focus here is to test the feasibility of applying automated methods to track mitochondrial motion using data generated by a robotic microscope.

One approach for the automated tracking of mitochondrial motion is to measure optical flow, for example by using the Lucas Kanade Motion Tracking Algorithm (LKMTA) (Gerencser and Nicholls, 2008). There are several excellent papers that have discussed its use and mathematical foundation (Lucas and Kanade, 1981; Jahne, 1997; Gerencser and Nicholls, 2008). In brief, optical flow is defined as the apparent motion of the brightness patterns in the image (Baker et al., 2011). While the full implementation of optical flow tracking algorithms is relatively complex because they incorporate methods for accommodating noisy data (Abràmoff et al., 2000; Gerencser and Nicholls, 2008), the underlying idea is straightforward (Fennema and Thompson, 1979). When an object moves the local brightness changes. The larger the change in brightness over time, the faster the motion. The strength of optical flow approaches is that they provide a relatively unbiased picture of mitochondrial movement from rates ranging from 0.01 to 2 μm/s (Gerencser and Nicholls, 2008). Nonetheless, they do not track the motion of individual objects over time.

A second major approach to the automated tracking of mitochondria is to use particle tracking (Chenouard et al., 2014). In brief this works by identifying a feature (e.g., a mitochondrion) at a particular time point, finding that feature in the next time point using a set of rules, and then generating a track that connects movement through time. There are currently several options for particle tracking including MTrack2, MTrackJ, Volocity, Imaris, wrMTrck, and Difference Tracker (Meijering et al., 2012). In a recent paper that compared the last four of these approaches with manual tracking of mitochondria (Bros et al., 2015), Difference Tracker (DT) compared fairly. In addition, it is relatively user friendly, free as an ImageJ plugin, produces concise output, is fast and can easily be incorporated into batch macros. For these reasons we were interested in testing its suitability for analyzing data acquired from a robotic microscope.

In terms of processing large datasets where the intent is to track the motion, there are three major problems that arise as the scale of the studies increases. The first is the obvious problem of processing time and whether simple, but fast approaches are capable of producing meaningful output from real data. The second is the development of methods to filter out spurious data that arise as the result of flicker in the illumination system, stage drift and focal drift. The final is the development of approaches that allow the convenient numerical processing and visualization of the motion of millions of objects.

To address these problems, we first compared the LKMTA and DT algorithms (DTA) with manual tracking using a simple test case where a single mitochondrion was in motion. This illustrated that these automatic tracking algorithms are both capable of detecting fast mitochondrial transport and shows what it appears as in terms of the outputs of the algorithms. Before moving to the problem of analyzing a large data set, we focused on the application of the algorithms to a single pair of movies. One that assessed motion before the application of nocodazole and a second following the application of nocodazole. As it is well known that nocodazole depolymerizes microtubules and in turn disrupts fast mitochondria transport (Samson et al., 1979; Morris and Hollenbeck, 1995; Ligon and Steward, 2000), this provided a direct demonstration that the DTA and LKMTA can detect the reduction in transport induced by nocodazole in a single set of movies. Using this example, we also explain how spurious data is filtered. With this foundation, we then use the LKMTA and DTA to analyze a large data set generated on a robotic microscope and examine the effects of nocodazole in the disruption of fast axonal mitochondrial transport in hippocampal and cortical neurons. We find that these approaches for measuring optical flow and single particle tracking are both feasible for use in a large-scale screen.

## Materials and methods

### Primary culture of hippocampal and cortical neurons and nocodazole treatment

Primary hippocampal and cortical cultures were prepared from the brains of embryonic day 18–21 CD1 mice (Jackson Laboratories). Housing, animal care and experimental procedures were consistent with the Guide for the Care and Use of Laboratory Animals and approved by the Institutional Animal Care and Use Committee of the Scripps Research Institute. Cells were plated at a density of 15–20 K per well on poly-D-lysine-coated (0.1 mg/ml) 96-well plates, according to procedures described previously (Liu et al., 2014). Briefly, isolated hippocampi and cortices were digested with 0.6 mg/ml papain in 1 × HBSS at 37°C for 15 min. Dissociated cells were plated in Neurobasal medium (Invitrogen) supplemented with 10% fetal bovine serum and penicillin/streptomycin mix and grown in Neurobasal medium supplemented with 2% B27 (Invitrogen), 0.5 mM glutamine, and penicillin/streptomycin mix at 37°C in 5% CO_2_. At 4 days *in vitro* (DIV4), one half of the media were removed and replaced with an equal volume of fresh media and the same protocol for medium change was performed every 4 days.

### Image acquisition using IN cell analyzer 6000

At DIV9, hippocampal and cortical cultured neurons were stained with 100 nM MitoTracker Green (Invitrogen, M7514) for 15 min at 37°C and then imaged under growth conditions (37°C, 5% CO_2_) using an IN Cell Analyzer 6000 (GE Healthcare Life Sciences) before and after nocodazole treatment (5 μM for 1 h.). Images were observed through a Nikon 60x/0.95, Plan Apo objective, Corr Collar 0.11–0.23, CFI/60 Lambda with an open aperture and 200 ms exposure at 488 nm excitation. During acquisition, laser focus was auto-adjusted so that high contrast images were acquired.

### Estimation of total mitochondrial number

To estimate mitochondrial number, images were stretched by a factor of 8 in x and y using TransformJ > Scale > quintic-B interpolation and the Find Maxima > Count function in FIJI was applied, after adjusting the noise tolerance, to find the total number of intensity maxima.

### Running the LKMTA and finding the optimal settings

To implement the LKMTA, the source code for the FlowJ plugin was downloaded from http://fiji.sc/FlowJ and modified to make two new functions: one called “Vary Parameters and Save” that systematically varies Σ*w*, Σ*s*, Σ*t*, and τ and applies those settings to a movie; and a second called “Compute and Save x y flow fields” that applies a given set of parameters to all of the frames in the movie. In both cases, the plugins save the x and y velocity maps which provide information about the local direction and magnitude of motion. In these maps, pixel intensity is high and positive in the x velocity map when there is rapid rightward movement and in the y velocity map when movement is upwards.

Since we are interested in the absolute velocity of the movements and are not concerned with their direction, we produce absolute velocity maps by squaring the pixel intensities of the x and y velocity maps, adding the pixel intensities of the two maps together and then taking the square root of the result. To quantify the motion, we then measure the histogram of the pixel intensity values in the absolute velocity maps. This generates a list of numbers that indicates how many pixels had a given level of intensity. Because pixel intensity is a function of local velocity, this provides an overview of the motion that is occurring in a movie. We call these velocity histograms.

### Optimization of settings

The settings for the spatial and temporal filters Σ*s* = 2 and Σ*t* = 2 were chosen based on a parallel analysis we conducted that indicated that these are the optimal values to use for quantitative assessment of motion tracking using the FlowJ plugin. We strongly caution that if Σ*s* is not equal to Σ*t*, the intensity of the flow fields will not be linearly related to velocity. Furthermore, if a value other than 2 is used for these variables the quality of tracking decreases. To determine the optimal settings for the window and noise variables Σ*w* and τ, we systematically varied Σ*w* from 0.1 to 1 in 0.1 increments and τ from 0.00001 to 5.24 where the initial value of τ was consecutively doubled 20 times. We then examined the output of velocity histograms to find the smallest value of Σ*w* that produced flow data (i.e., 0.3) and largest value for τ (i.e., 0.02) that fully suppressed noise generated in regions not containing mitochondria.

### Processing of image data

This provides an overview of the steps that were used for the processing of the data. In general all of the processes were executed using batch macros in ImageJ and the resulting files were saved at each step.

1. Import the movie file into ImageJ.
2. Bin images by 4x (Image > Adjust Size > 512 × 512 > Average when downsizing > Interpolation: Bilinear). Based on the results from a parallel study, this improves the tracking of rapidly moving mitochondria.
3. StackReg each 50 frame movie (Plugins > Registration > StackReg > Transformation: Rigid Body) to correct for stage drift.
4. Stretch images by 2 on the time axis (Plugins > Transform > TransformJ > Scale > x-factor for scaling: 1, y-factor for scaling: 1 z-factor for scaling: 2 > Interpolation scheme: quintic B-spline). Based on a separate analysis, this also improves the tracking of rapidly moving mitochondria.
5. Determine the optimal values for the FlowJ plugin. Crop out a small section of a movie that has the motion of interest and is representative of the data set. Directly measure the motion of interest using a kymograph or other trusted approach to establish “ground truth” flow. As an additional control, it may be helpful to construct a parallel movie where the motion of interest is absent. Set Σ*s* = 2 and Σ*t* = 2, systematically vary τ and Σ*w* and produce x y flow maps. First examine how the output varies as a function of Σ*w*. Pick the smallest value that generates a clear flow field for the moving object, then for that value of Σ*w* examine the output as a function of τ. Find a value for τ that fully suppresses flow associated with image noise, but does not suppress the flow fields associated with mitochondria. A straightforward way to do this is to examine the flow fields in a region of an image that both contains and lacks mitochondria. Take these values and conduct spot tests on different movies in the data set to ensure that they produce reasonable output.
6. In FlowJ use the optimal settings determined in the previous step to produce x y flow maps for each time point in the data series. This was done by combining individual movies into large single stacks (using File > Import Image Sequence) and running a modified version of the FlowJ plugin using the “Compute & Save x y flow fields” setting.
7. Crop images to remove edge artifacts (i.e., 512 × 512 to 450 × 450 pixels) using Image > Adjust Canvas Size. Log transform the images using Process > Math > log and replace zeros with NaN.
8. Open the cropped, log transformed, zeros set to NaN absolute velocity histograms as a single stack and run the StackHistogramLister macro using the settings of: Number of bins = 140, Histogram Min = −10, and Histogram Max = 4. Save the results as a text file. Because the pixel intensities in the images are log transformed, this measures the output over the range of 0.000045–54.6 p/f.
9. Open the text file as an image using File > Import > Text Image, then using the Montage to Stack and Make Montage plugins convert this to a velocity histogram montage that has the individual velocity histograms aligned for each time point along the x–axis.
10. Normalize the output of the velocity histograms by dividing them by the average value of the histogram. Remove time points where there is a large change in the velocity distribution over the three images by examining the standard deviation of three adjacent velocity histograms. Produce a two-dimensional “flow table” which has time on the y-axis and the sample number on the x-axis. In the flow table, pixel intensity is equal to the ratio of flow above a threshold above a defined value (e.g., 0.25 μm/s).
11. Save the flow table as a text image and open it in Excel. To make it easier to process the data in Excel, convert zeros to NaN before saving the flow table. This prevents the problem of averaging time points that lack data into the estimates of flow.

### Processing data with difference tracker

When using Difference Tracker steps 1–4 are the same as above.

5. The images are then cropped to 400 × 400 pixels to remove edge artifacts caused by StackReg.
6. The movies are normalized using the “Enhance Contrast” ImageJ plugin with the settings: Saturated pixels: 5%, Normalize and Equalize histogram.
7. The movies are converted to an 8-bit pixel depth.
8. The movies are processed using the Difference Filter plugin using the settings of Minimum Difference: 20 and Difference Frame Offset: 4. This creates and saves a text file that contains descriptive data and thresholded movies where the stationary mitochondria are removed.
9. The movies produced by the Difference Filter are processed using the Mass Particle Tracker with the settings Minimum tracked intensity: 20, Minimum feature size: 2, Initial flexibility: 10, Subsequent flexibility: 5, and Min track length: 4. This creates and saves a second text file that contains additional descriptive data and movies with tracks overlaid on the path of moving mitochondria.
10. The text files generated by the Difference Filter and Mass Particle Tracker are opened in Excel and processed to analyze the transport statistics.

### Statistical analysis

To determine if the level of transport in a particular sample treated with nocodazole was statistically different than in the controls, the data were analyzed by One-Way ANOVA in Minitab using Dunnett's test. For this analysis, the control samples for the hippocampal neurons and the cortical neurons were pooled separately. Data from each of the treated samples was then compared against the corresponding pooled control for the hippocampal and cortical neurons.

## Results

### Illustrating the use of the LKMTA to track the movement of a single mitochondrion

**Figure 1:**
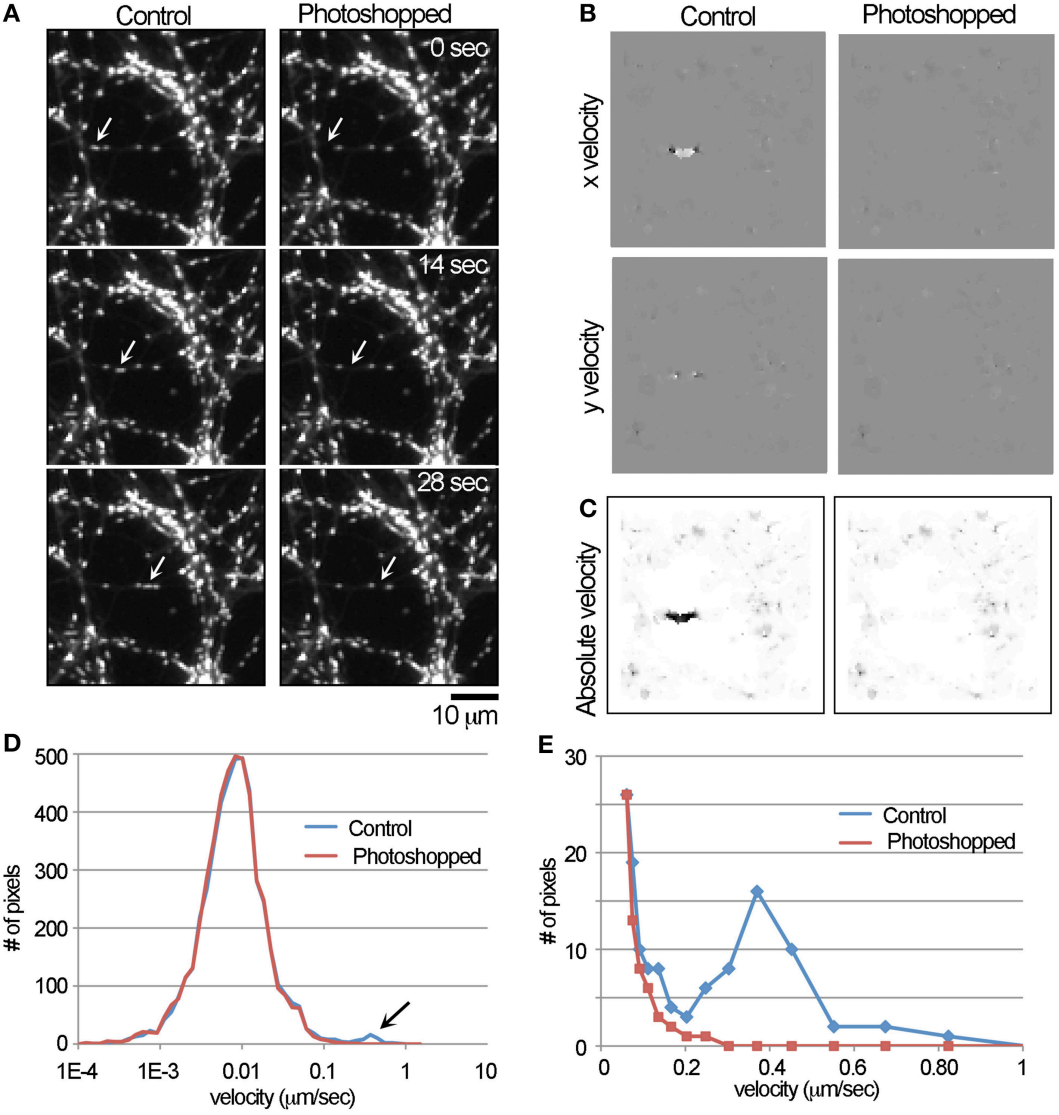
**Detection of the movement of a single mitochondrion with the LKMTA. (A)** Panels from a time-lapse movie where a single fast transported mitochondrion was digitally removed in Photoshop. The original movie is labeled as the control and the arrows indicate the position of the moving mitochondrion; bar = 10 μm. **(B)** The x and y velocity maps of the control and Photoshopped movies; a cluster of bright pixels in the control x velocity map indicates rightward motion. **(C)** The absolute velocity maps generated from the x and y velocity maps; color is inverted. **(D)** The full velocity histogram shows that the local velocity for most of the pixels is approximately 0.01 μm/s and there is a small peak in the control plot in the range between 0.1 and 1 μm/s. **(E)** Re-plotting a velocity histogram from the range of 0.05–1 μm/s illustrates the tracking algorithm produces a peak centered at 0.4 μm/s.

To confirm that the LKMTA as implemented in FIJI as the FlowJ plugin (Abràmoff et al., 2000, 2004) is capable of detecting fast mitochondrial transport (Gerencser and Nicholls, 2008), we analyzed a region of a larger movie where only one out of a total of approximately 200 mitochondria were undergoing fast transport. To use the LKMTA, one takes a set of time-lapse images and defines the size and shape of the spatial filter Σ*s*, the temporal filter Σ*t*, the window function Σ*w*, and the noise threshold τ. The plugin then produces x and y velocity maps where local pixel intensity is equal to the estimated optic flow in units of pixel/frame (p/f). For example, if an object is moving at a velocity of 1 p/f, it will create a “hot spot” in a velocity map with pixel intensity values of 1. Based on a parallel analysis we conducted to find the best approach to pre-filtering movies to optimize motion tracking, we first binned the movie by a factor of 4 in the spatial dimensions, used the StackReg function in ImageJ to remove global drift in the movie and stretched it by a factor of two on the time axis. We then rotated the movie so that the mitochondrion moved solely along the x-axis and cropped it to 100 × 100 pixels in x and y and 18 frames (i.e., 28 s) in time. As a test of the capabilities of the tracking algorithm, we also prepared a movie where Photoshop was used to digitally remove the moving mitochondrion (Figure 1A). This was done by converting the movie into a montage, exporting it into Photoshop and using the clone stamp tool to digitally remove the mitochondrion. During this process care was taken to ensure that the position and intensity of stationary mitochondria were unchanged. Because the difference between the movies was the removal of one mitochondrion, we could be reasonably confident that differences in the output of the LKMTA could be attributed to the movement of that mitochondrion.

With both the control and Photoshopped movies (See Supplementary Movies 1, 2), we ran the FlowJ plugin using the settings: central slice = 9; Algorithm = Lucas & Kanade; Gradient method = Gaussian deriv; Σ*s* = 2; Σ*t* = 2; τ = 0.02; Σ*w* = 0.3; and Regularization = Gaussian 1D. These were chosen based on an extensive and systematic analysis of how these parameters impact the output. This produced output in the form of x and y flow maps (Figure 1B) where pixel intensity is equal to the local flow field in the units of p/f. In the flow maps, the fast transported mitochondrion produced a discrete cluster of bright pixels in the control image, which was absent in the Photoshopped version. In addition, regions containing stationary mitochondria produced small flow fields (which appear as a faint roughness) and areas that lacked mitochondria produced no output (which appear flat). The x y velocity maps were then converted to absolute velocity maps, where pixel intensity scales with velocity irrespective of direction (Figure 1C). While is it clear that there is a local region consistent with flow, an initial challenge was developing a numerical approach to measuring that motion. The problem is that out of the 10,000 pixels in the 100 × 100 pixel images used for this analysis, pixel intensity is only changing in approximately 40 pixels. Our solution was to first log transform the absolute velocity map and then to generate a histogram of the pixel intensities (Figure 1D) over the range of -10 to 4. Keeping in mind that a pixel intensity of 1 equals a velocity of 1 p/f, the log transformed range covers velocities from 0.000046 to 54.6-p/f, which is 0.00001 to 13.5 μm/s. Based on the time and spatial dimensions of the movie this was then converted into velocities. Examining the graph and noting the log scale on the x-axis, the velocity profile has a log normal distribution with a peak at approximately 0.01 μm/s. This is consistent with the observation that mitochondria are for the most part stationary and undergo small back and forward movements. In addition, there is a small peak between 0.1 and 1 μm/s that is present in the control, but not the Photoshopped profile. To examine this more closely, we re-graphed the data over the range of 0.05–1 μm/s. In that graph, it is clear that the number of observations drops steeply from 0.05 to 0.2 μm/s and that there is a broad peak at approximately 0.4 μm/s in the unPhotoshopped plot (Figure 1E). This peak indicates the LKMTA can detect the motion of a mitochondrion moving by fast transport.

### Comparison of tracking by manual point selection, kymographs, LKMTA, and DTA

**Figure 2:**
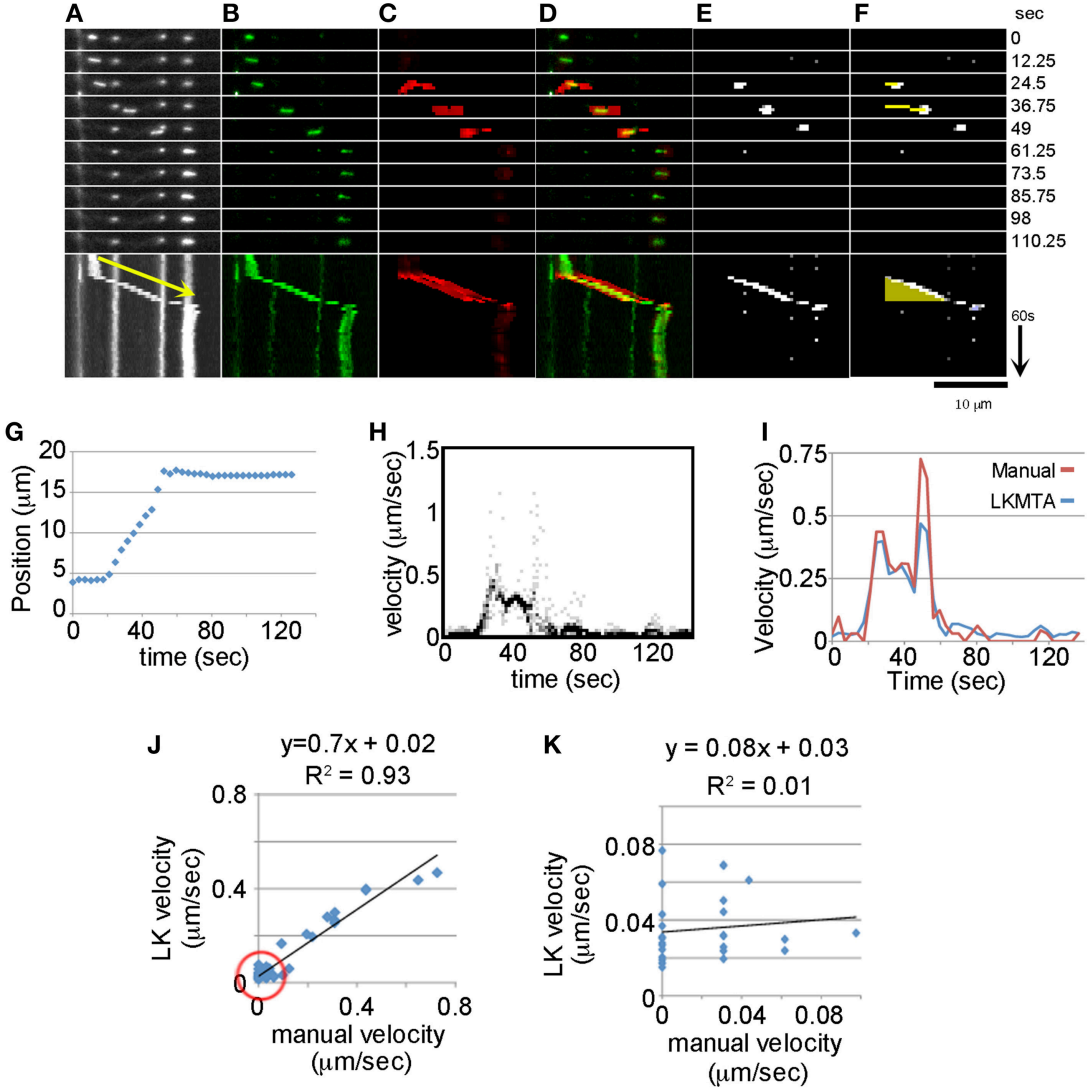
**Comparison between Manual Tracking, the LKMTA and the DTA suite of plugins. (A)** Fluorescent images of mitochondria with a kymograph below. The yellow arrow corresponds to a velocity of 0.39 μm/s. **(B)** Background subtraction removes stationary mitochondria and highlights a mitochondrion that initiates movement and then pauses. **(C)** Flow maps generated by the LKMTA of the background-subtracted image. **(D)** Overlay of the moving mitochondrion and the flow maps show their overlap in the still frames and kymograph. **(E)** The output from the Difference Filter. **(F)** Output from Mass Particle Tracker, the yellow line is the track of the moving mitochondrion and it appears as a yellow triangle in the kymograph. **(G)** Results from manual tracking by point selection. **(H)** The velocity histograms as a function of time generated from the LKMTA flow maps. **(I)** A comparison of the estimated velocity using manual tracking and the LKMTA. **(J)** The correlation between the manual and LK estimates of velocity over the full velocity range. The red circle shows the region analyzed in the next panel. **(K)** The correlation over the velocity range of 0–0.1 μm/s.

To compare the LKMTA with manual tracking and single particle tracking by the Difference Tracker suite of plugins, we took the data from the same movie as in Figure 1, but cropped and over a longer time period (Figure 2). In the movie (Figure 2A), the mitochondrion is initially stationary, but then moves at a relatively constant velocity and pauses. Using manual analysis by kymograph, the mitochondrion had a run distance of 13.8 μm, a run time of 35 s and thus a velocity of 0.39 μm/s. To track its motion manually by point selection, we marked its position in each frame using the measure function in ImageJ; the output is shown in Figure 2G. Focusing on the average change in position over the change in time during the period when the mitochondrion moved at > 0.1 μm/s the average velocity was 0.39 ± 0.18 μm/s (ave ± sd, *n* = 10 time points). This suggested that the manual estimates of velocity and the LKMTA estimate of velocity (Figure 1) are similar.

To directly compare motion tracking by the LKMTA with manual tracking through the time series, we needed to remove the stationary mitochondria from the movie. The reason is that they generate flow fields, albeit of a small magnitude, that would complicate the analysis of motion. Because digital removal of mitochondria, as in Figure 1, has the potential to generate image artifacts, we used the alternative approach of removing the stationary mitochondria from the movie by background subtraction. This was done by doing an average Z projection of the entire movie and then subtracting that from each image in the movie (Figure 2B). As a result the “stationary” mitochondria were for the most part removed from the time series. While faint traces are still seen in the individual frames and kymograph in Figure 2B, because there is a background noise setting in FlowJ (i.e., τ) that suppresses the generation of flow fields of low intensity objects, the flow fields of the faint objects were suppressed. We then ran FlowJ using the same settings as in Figure 1 to produce flow fields for each time point (Figure 2C). Examining the kymographs as the mitochondrion in green begins moving, a strong flow field in red appears, which greatly weakens as it pauses (Figure 2D). To estimate velocity for each time point, we generated histograms of the velocities for each pixel and plotted them as a function of time (Figure 2H). This 2D picture is in essence a series of graphs like those shown in Figure 1E, where the intensity of the dots is proportional to the number of pixels with a given intensity. To calculate the velocity for each time frame we took the average of the velocity histograms at each time point and plotted these in Figure 2I. Comparing the velocities estimated by the LKMTA and manual point selection, there is a good correspondence (Figure 2I). Focusing on the time points in Figure 2I where velocity is > 0.1 μm/s, the average estimate for velocity from the LKMTA is 0.34 ± 0.05 μm/s (ave ± sd, *n* = 10). Examining the correlation between the velocities over the full range of the motion there is a strong correlation with an *R*^2^ of 0.93, nonetheless the slope is 0.7 instead of 1. This indicates that the LKMTA underestimates velocity as compared to the manual tracking and this is most apparent in the divergence of the velocity estimates at approximately 50 s in Figure 2I.

While manual tracking provides an accurate assessment of rapid mitochondrial movement, it has the limitation of only measuring movements that are greater than 1 p/f. In contrast, the LKMTA is capable of measuring sub-pixel movements (Bouguet, 2001). To examine the correspondence between manual tracking and the LKMTA for very slow movements, we plotted the relationship between movements in the velocity range between 0 and 0.1 μm/s (Figure 2K) and found no correlation (*R*^2^ = 0.01). While the lack of correlation is expected based on the known limitations and strengths of manual tracking and the LKMTA, we include this result to illustrate that one reason different approaches to measuring mitochondrial transport give differing results is that they measure different ranges of motion. In this particular case, manual tracking is arguably more precise than the LKMTA in quantifying rapid movement, but is unable to measure sub-pixel motion. In contrast, the LKMTA detects a larger range of motion, that extends to sub-pixel movements, but has a lesser degree of precision.

As a means to compare how well the LKMTA tracks motion as compared with another automated mitochondrial tracking algorithm, we repeated this analysis (Figures 2E,F) using the Difference Tracker suite of plugins in ImageJ (Andrews et al., 2010). The first, called the Difference Filter, uses a fairly sophisticated background subtraction algorithm that compares the local pixel intensity in the frame of interest to pixel intensity in a frame given by a specified time offset. It then thresholds the image using a noise filter to generate time-lapse series that only contains moving mitochondria (Figure 2E). In addition, it generates descriptive information that includes estimates of the number of stationary and transported mitochondria. Using the output of the Difference Filter (Figure 2E), the second plugin, called the Mass Particle Tracker, then identifies clusters of bright pixels (i.e., features), and assigns tracks to those that appear as lines (Figure 2F). It does this by initially looking for the nearest feature in the following frame. Once a track is started, the observed motion between the two frames is used to generate a prediction of a feature in the next frame. In the plugin, parameters that define the size of the pixel cluster and the parameters for tracking them are set. The tracking occurs by a set of rules that ensures each particle is associated with a single track and longer tracks have priority over shorter ones in terms of the new feature assignment. These tracks are colored yellow for objects moving from left to right and blue for those moving from right to left. After all tracks are defined, short tracks can be removed in the software to decrease what may be spurious noise. From the tracks, descriptive data are generated about track number, direction, duration, and velocity (Andrews et al., 2010; Bros et al., 2015). Figure 2E shows the output of the Difference Filter when it was run with the settings of minimum difference = 20 and difference frame offset = 4. Figure 2F shows the output of the Mass Particle Tracker when run with the settings of Minimum tracked intensity = 20, Minimum feature size = 2, Initial flexibility = 10, Subsequent flexibility = 5, and Min track length = 4. In the panels, a yellow track is seen that follows the mitochondrion (Figure 2F). Because the track becomes longer with each frame and disappears when the track stops getting longer in the kymograph below it appears as a yellow triangle. Looking at the output of the Mass Particle Tracker, the mitochondrion had an average estimated velocity of 0.32 μm/s with a run time of 24.5 s. As with the LKMTA, this is an underestimate of velocity as compared with the manual estimates of 0.39 μm/s. Careful examination of the data indicates that this lower estimated velocity occurred because the track was lost at approximately the 50 s time point when the mitochondrion moved at a rate of roughly 0.6 μm/s. Together, this demonstrates how the LKMTA and DTA are used to track mitochondria and shows that while they are less precise than manual tracking they give similar overall estimates of fast mitochondrial transport velocity.

### Illustration of the use of the LKMTA and DTA to measure the reduction in fast mitochondrial transport induced by nocodazole in a single pair of movies

**Figure 3:**
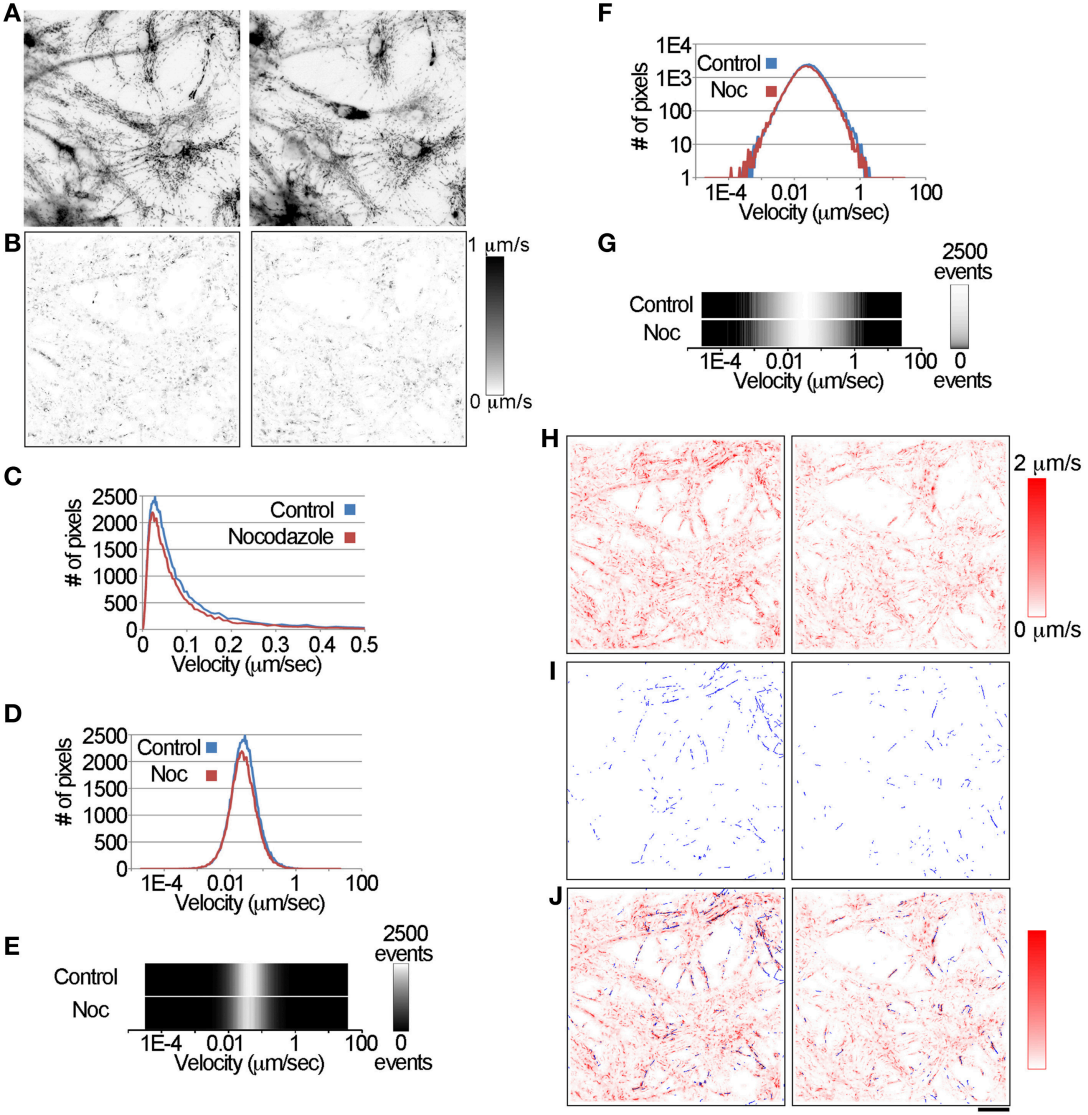
**Using the LKMTA and DTA to measure the reduction in fast mitochondrial transport in a single pair of pre-drug/post-nocodazole movies. (A)** A set of images from a single well, before and after application of nocodazole. **(B)** The absolute velocity maps for the control and nocodazole treated samples at a single time point. The bar to the side shows the scaling between pixel intensity and local velocity. **(C)** The velocity distributions for control and nocodazole treated samples generated from the absolute velocity maps. **(D)** The same data plotted on a log scale. **(E)** A pictorial representation of the graph in **(D)**, where pixel intensity is equal to the number of pixels. The bar to the side shows the scaling. **(F)** The same data again, but plotted on a log-log graph. **(G)** A pictorial representation of the data in **(F)**. **(H)** A maximum Z-projection of all of the absolute velocity maps in the time series. **(I)** A maximum Z-projection of all of the tracks produced by the Mass Particle Tracker plugin in the time series. **(J)** Overlay of the output of the LKMTA and Mass Particle Tracker algorithm in red and blue. There is a similar overall reduction in the flow maps and the number of tracks, as well as a correspondence between their distributions; bar = 25 μm.

**Figure 4:**
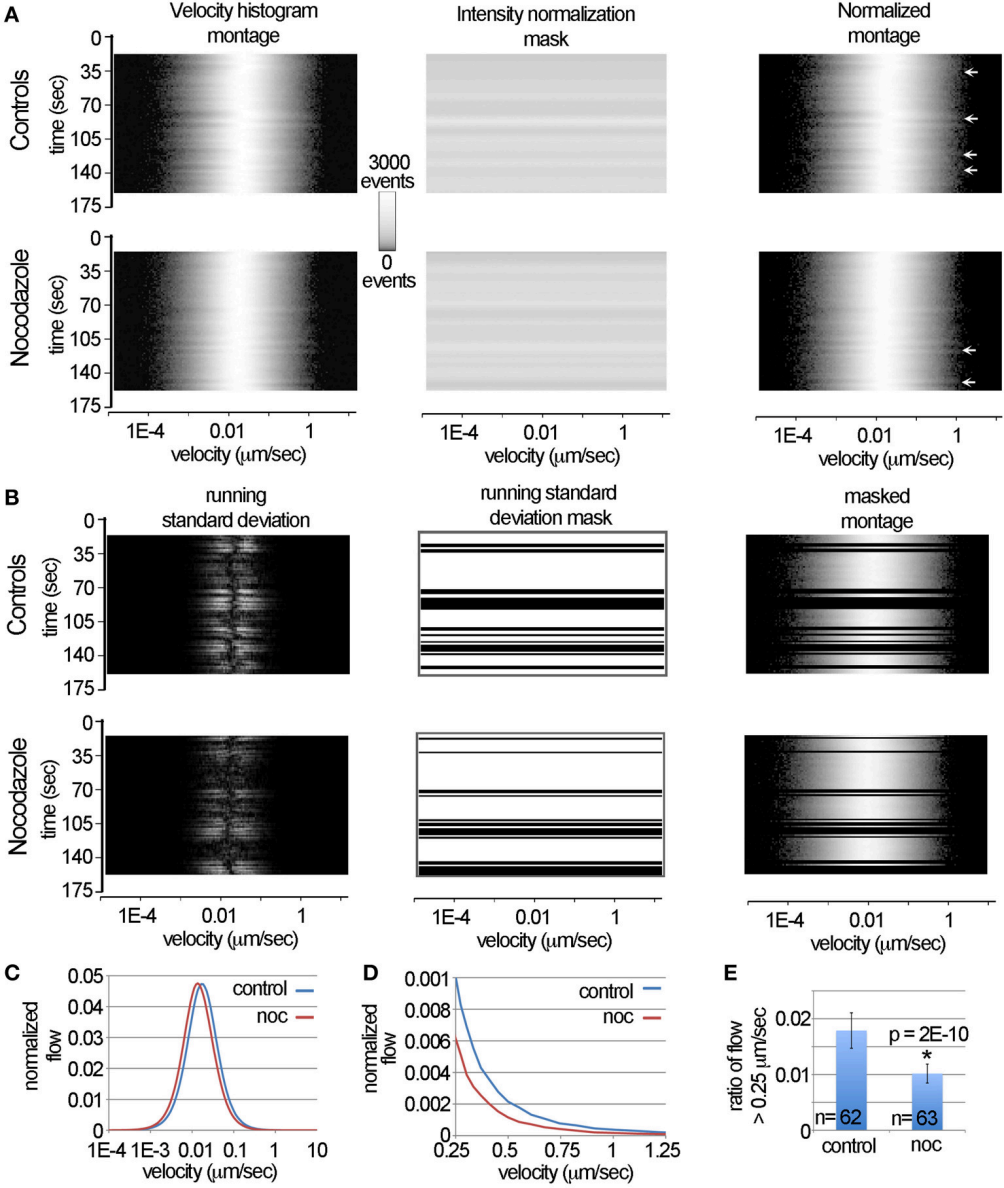
**Automated removal of spurious motion using the LKMTA. (A)** Illustration of the steps used to normalize the velocity histograms produced by the LKMTA. **(B)** Illustration of the steps used to remove transient changes in the velocity profiles caused by stage drift and illumination flicker. **(C)** The plots of the velocity histograms of the pre-drug and post-nocodazole conditions. **(D)** The velocity profiles over the velocity range characteristic of fast mitochondrial transport. **(E)** The ratio of movements with velocities in the range of 0.25–4 μm/s for the control and nocodazole treated samples (ave ± sd).

Having demonstrated that the LKMTA and DTA are capable of detecting mitochondrial motion under tightly controlled conditions, our next question was whether they could be used to measure changes in fast mitochondrial transport under conditions relevant to the implementation of a large-scale screen. As an intermediate step, before discussing the screen we focus on the results of a single pair of movies. In particular, this figure is needed to explain the way that we present transport data over time, which is shown in the next figure. As a way to disrupt fast mitochondria transport, we added the microtubule disrupting drug nocodazole to the cultures because it is well accepted to disrupt fast mitochondrial transport (Samson et al., 1979; Morris and Hollenbeck, 1995; Ligon and Steward, 2000). In our experiments, neurons are cultured in 96-well plates and labeled with MitoTracker Green to visualize the mitochondria. For each well a 175 s movie consisting of 50 frames at 3.5-s intervals were acquired. After each plate was scanned, nocodazole was added at a concentration of 5 μM, incubated for 1 h and the plate was rescanned. Figure 3A, shows typical images of the distribution of labeled mitochondria from a single pre-drug and post-nocodazole pair of hippocampal neurons. One may note that the overall distributions of mitochondria/neurons in the images are similar. This is because the data were collected from the same well. As detailed above, the movies were preprocessed and the motion was analyzed using the same settings as described above. In addition, we tested and confirmed that photo-bleaching was below detectable limits in these experiments (Supplementary Figure 1 and Supplementary Discussion). To illustrate the output of the LKMTA and the DTA for these two movies, we first show the absolute velocity maps generated by the LKMTA for a single time point (Figure 3B). In this figure, pixel intensity is proportional to the local velocity, with darker pixels corresponding to regions where motion was faster. The intensity bar to the side of Figure 3B shows the mapping between intensity and velocity. The visual impression from the absolute velocity map is that the average velocity of movement is higher in the control than the nocodazole treated sample. Plotting the number of pixels as a function of velocity (Figure 3C) confirms this impression. One issue we found in processing and presenting the data generated by the LKMTA is that it generates a large number of measurements at very small velocities. To visualize these slow movements, we also the plot the same data using a log scale (Figure 3D). Here again the decrease in transport associated with nocodazole treatment is seen. In moving to an analysis of how the velocity distribution changes over time, a challenge was in developing a way to present thousands of graphs. Our approach to this problem was to export the list of numbers into ImageJ as a text image to create a visual representation (Figure 3E). In doing so for a single time point, a one pixel thick line is generated where pixel intensity is equal to the # of pixels plotted on the y-axis in Figure 3D. Because a one pixel thick line is difficult to see, we then stretched it by a factor of 50. One aspect of this presentation that is problematic is that transport in the velocity range of 0.1–1 μm/s is difficult to see in the picture. To better illustrate movement in this range, we plot the same graph as in Figure 3D, but now also using a log scale on the y-axis. Likewise, we take the image in Figure 3E and apply the Math > Log function in ImageJ. This is the same data as in Figure 3C, but now in a visual log format. If we stack a series of images like this from different time points, it allows a complete profile of changes in the transport profile over time. Below in Figure 4, we call this a velocity histogram montage and use this format to show how we process the data to remove spurious data that results from changes in illumination or stage drift.

### Using the DTA plugins to measure the reduction in transport induced by nocodazole

To compare and contrast the LKMTA and DTA, we first generated absolute velocity maps for every time point in both the control and nocodazole treated samples generated using the LKMTA and did a maximum Z-projection (Figure 3H). The bar that goes from red to white on the side shows the mapping between velocity and pixel intensity. These images show at a glance where the LKMTA reported fast mitochondrial transport in both movies. We then analyzed this data using the DTA suite of plugins. To show the output of the tracks produced by the Mass Particle Tracker, the output of the plugin was Z-projected for all time points in the movie (Figure 3I). Because the yellow tracks produced by the MPT were difficult to see, we converted them to blue and overlaid this on the Z-projected output from the LKMTA (Figure 3J). The overall alignment of the flow fields and tracks indicates that the LKMTA and the DTA are measuring similar movements. The smaller number of tracks, as compared to flow fields, was expected as the LKMTA measures and reports all motion, whereas the DTA only produces tracks when an object undergoes consistent movement over at least four time points. The similar reduction of tracks and flow fields following nocodazole treatment (Figure 3J) indicates that the LKMTA and DTA are both measuring a similar reduction in transport that occurred as the result of microtubule depolymerization. While analysis of the tracks here gives a useful visualize check, the strength of the DTA suite of plugins is that they produce concise numerical summaries of motion (Table 1). Examining the output of these plugins, there was a relatively small increase in the Static Intensity for the nocodazole treated samples and a large decrease, by approximately one half of the Moving Intensity. In addition, there were approximately 50% reductions in the Moving Count, Count Percentage Moving, Total Track Count and Average Particle Track per frame. These results illustrate the numerical output of the DTA plugins and indicate that they are capable of quantitatively assessing the reduction in fast transport induced by nocodazole.

**Table 1:** **Combined output of the Difference Filter and Mass Particle Tracker plugins showing the raw results and the ratio of the results for the data in Figure 3**.

**File name**	**Control**	**Nocodazole**	**Nocodazole/Control**
Static Intensity	2334480	2551856	1.09
Moving Intensity	6213	2796	0.45
Intensity Percentage Moving	0.28	0.12	0.43
Static Count	45767	48047	1.05
Moving Count	142	73	0.51
Count Percentage Moving	0.32	0.16	0.50
Total Track Count	411	240	0.58
Average Particle Count Per Frame	22	13	0.59
Average Track Duration	5.17	5.21	1.01
Average Volume Per Particle	4.5	4.1	0.90
Average Speed Per Track	1.03	0.85	0.83
Average Max Speed Per Track	2.2	1.9	0.85

### Using the LKMTA to filter imaging artifacts and to measure the reduction in transport induced by nocodazole

A common issue in generating time-lapse data, be it manually or by robotic microscope, is that stage drift and image flicker degrades the quality of the movies. When analyzing such movies by hand, it is relatively easy to ignore a few “bad” frames. For example, if data are missing at one time point in a kymograph it is straightforward to extrapolate through this time point. Nonetheless automated routines do not have procedures in place to remove such problems. With the goal of removing transient imaging artifacts that occur over a few time points, we developed a novel means of identifying problematic time points based on large changes in the overall velocity distribution profile. The underlying chain of logic is that our movies are acquired at intervals of 3.5 s for a period of less than 3 min. As this is a relatively rapid rate of acquisition over a short time frame, the expectation is that the velocity histograms, such as shown in Figure 3, would be very similar going from time point to time point. Furthermore, because approximately 95–99% of the mitochondrion are essentially stationary, one would expect that there should be very little variation in the overall velocity distribution from time point to time point. This is to say that the most likely reason that the average velocity of thousands of mitochondria would shift from 0.01 to 0.1 μm/s between two time points 3.5 s apart is that the stage moved or the illumination changed. With this in mind, we reasoned that large changes in the profiles of the velocity distributions going from one time point to the next might be useful for identifying and removing imaging artifacts in an automated manner.

One issue in developing and explaining approaches to filter the data in this way is that it is difficult to concisely show how the overall velocity distribution of mitochondria change over time. Our approach to this problem, described in part in Figure 3 is to plot out the graphs of velocity distributions as pictures. In these pictures, time is on the y-axis, velocity is on the x-axis and pixel intensity corresponds to the number of measurements. We call these pictures velocity histogram montages. To illustrate, we show these in Figure 4A for the full time series of the control and nocodazole treated data sets introduced in Figure 3. To convert the velocity histograms into a format where the relative, as opposed to the absolute, levels of transport can be calculated, we normalized the raw data in the velocity histogram montages by dividing them by the average pixel intensity value for each time point (i.e., the intensity normalization mask). This does not change the overall shape of the curves, it simply adjusts them so the area under the curves are identical across time. The main reason this is important is that it factors out the total number of mitochondria in a sample, so that instead the overall velocity profile can be considered.

In examining the normalized montages, in general the velocity profiles appear relatively similar over time, nonetheless occasionally there are somewhat subtle shifts that appear as “ripples” (see arrows in Figure 4A). On examining the raw movies, these ripples correspond to transient changes in illumination (i.e., flicker) and stage drift that was not fully corrected by image registration using StackReg. Our solution to identifying these was to calculate the running standard deviation of the velocity histograms over three time points. To do this, we transformed the normalized velocity histogram montages to a stack with three identical images. We then shifted the image on the top of the stack backwards by one time point and the image on the bottom forward by one time point. Next, we Z-projected the stack using the standard deviation function in ImageJ. This created an image where the pixel intensity is equal to the running standard deviation over three time points.

To generate the running standard deviation mask to filter the data, we summed the standard deviations for each time point, which created a single column of data. This column was then stretched to create a new image that was then thresholded. By multiplying this by the normalized velocity histogram montage, transient changes in the velocity histograms were removed in a consistent and automated manner (Figure 4B). As can be seen by comparing the normalized montage (Figure 4A) with the masked montage (Figure 4B), the ripples in the velocity histogram have been removed.

To measure the velocity profiles, the processed velocity histogram montages were saved as text files and opened in Excel. The average normalized velocity distributions were then calculated (Figure 4C). They indicate that nocodazole produces an overall reduction in the velocity distribution, which appears as a leftward shift in the nocodazole treated velocity profile. To more closely examine how nocodazole affected fast mitochondrial transport, we then plotted the velocity profiles over the range of 0.25–1.25 μm/s (Figure 4D). This showed that nocodazole on average reduced fast transport by approximately 40%. For example the normalized flow at 0.25 μm/s for the control condition was 0.001 and in the nocodazole condition it was 0.0006. To measure the effect that nocodazole had on the ratio of flow with a velocity > 0.25 μm/s, we calculated the average of the pixel counts between 0.25 and 54 μm/s for all of the time points in each movie (Figure 4E). Because some of the time points were removed using the running standard deviation mask the number of observations equals 62 and 63 as opposed to 80. A *t*-test indicated that nocodazole significantly (*p* = 2E-10) reduced transport by 43%. In terms of comparison, the Difference Filter plugin indicated that the number of fast transported mitochondria was reduced by 42%. Together, this demonstrates that the DTA and the LKMTA provide similar estimates in terms of the relative reduction in transport in a single sample and provides a detailed explanation of how the data were processed for the large-scale screen described in the next section.

### The application of the LKMTA to a large data set

**Figure 5:**
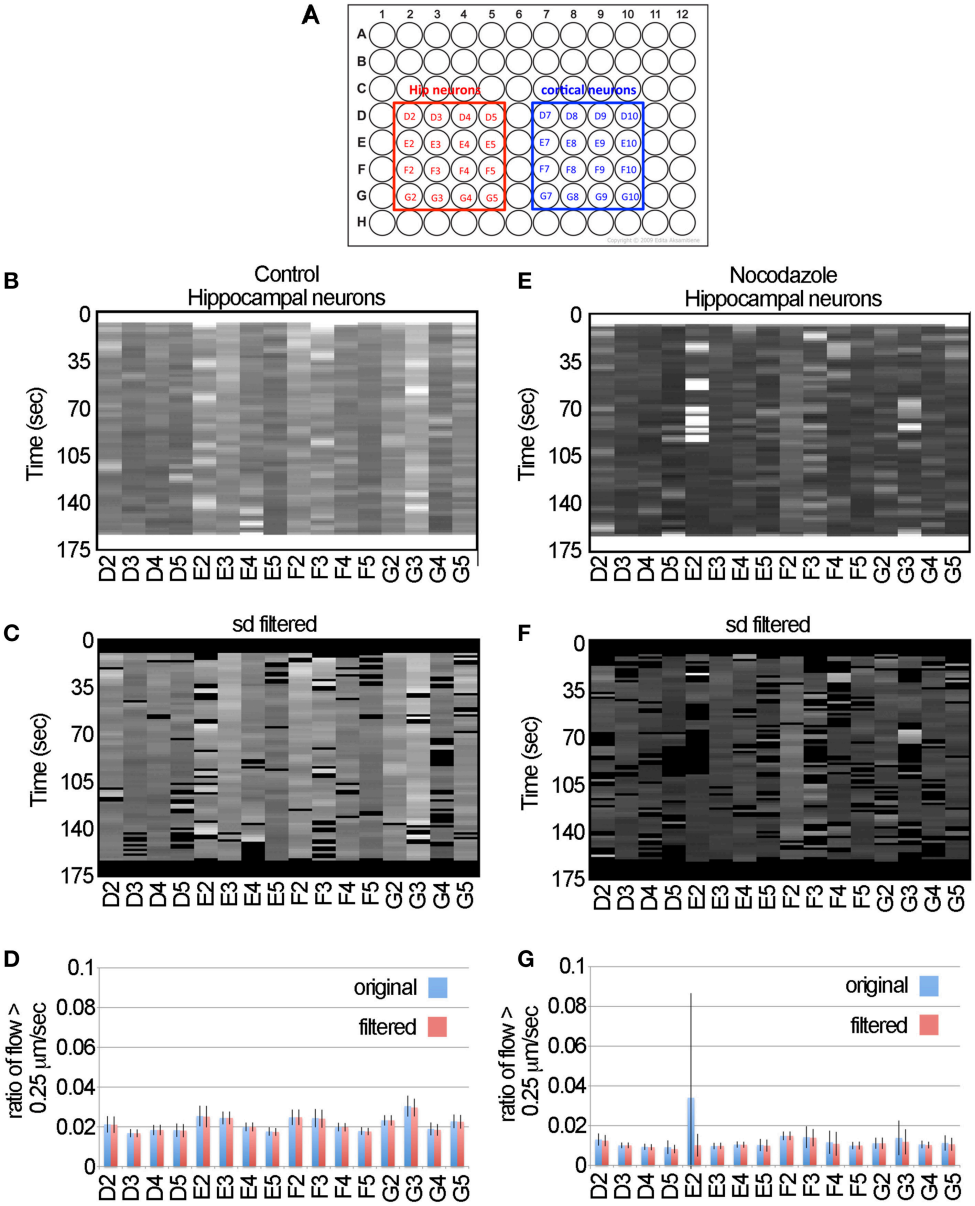
**The LKMTA consistently identifies reduced transport following nocodazole application in hippocampal neurons. (A)** Map of the 96-well plates. **(B)** Unfiltered flow table for control hippocampal neurons. **(C)** Filtered flow table for control hippocampal neurons. **(D)** Average flows for each sample, based on the unfiltered and filtered analysis of the control hippocampal neurons. **(E)** Unfiltered flow table for nocodazole treated hippocampal neurons. **(F)** Filtered flow table for nocodazole treated hippocampal neurons. **(G)** Average flows for each sample, based on the unfiltered and filtered analysis of the nocodazole treated hippocampal neurons.

In moving to large data sets the primary concerns are developing automated approaches for collecting the data, ensuring the data can be processed in a reasonable amount of time and developing statistical approaches for identifying significant changes in transport. To collect the data, we used the IN Cell Analyzer 6000 imaging system (Mei et al., 2012). This is a line scanning confocal microscope equipped with a 60x NA 0.95 objective and autofocus capabilities. It acquires images using a large field-of-view sCMOS camera (2048 × 2048 pixels) and an automated stage. Given the magnification of the objective and the pixel size of the camera, the pixel dimensions are 0.108 μm/pixel, which is approximately 3-fold greater than the conservative theoretical maximum resolution given by the Rayleigh criterion for green light (i.e., 341 nm = 0.61 532 nm/ 0.95 NA) (Ram et al., 2006). For our large-scale experiments we collected 64 movies, the first 32 were pre-drug control and the second 32 were acquired by rescanning the same wells following treatment with 5 μM nocodazole. To test if the data generated by the system produced consistent results in different types of neurons, half of the neurons were hippocampal and the other half were cortical (Figure 5A).

The detailed steps for processing the data are described in the Materials and Methods section. In brief the movies for the control and nocodazole treated data sets were opened and the x y flow fields for each time point was generated using the settings noted in Figure 1. In terms of processing time, this took approximately 1 h (i.e., about 1 s per frame) on a MacBook Pro with a 2.5 GHZ Intel Core i7 for the 64 movies. The flow maps were then converted to masked normalized velocity histogram montages and then the sum of the pixel values that had a velocity greater than 0.25 μm/s for each time point were calculated to generate flow tables (Figures 5B,C,E,F). These show at a glance the ratio of the total flow with a velocity greater than 0.25 μm/s as a function of time for each sample. Visually they indicate that levels of fast transport were greater in the control conditions than in the nocodazole treated samples and serve as a means to assess the quality of the data.

To illustrate the effect of filtering the data using the standard deviation mask, we have included the unfiltered flow tables (Figures 5B,E), the standard deviation filtered flow tables (Figures 5C,F) and graphs of the average and standard deviation filtered data for the full data set (Figures 5D,G). For most of the samples, filtering the data had a negligible effect on the measured flow. For example, in the control data set of the hippocampal neurons the average ratio of flow with a velocity > 0.25 μm/s for the unfiltered and filtered data sets was 0.0215 ± 0.0037 and 0.0213 ± 0.0037 (ave ± sd, *n* = 16 for each), likewise there was no significant difference when comparing these two groups by Student's *t*-test (*p* = 0.89). Nonetheless, in the flow table for nocodazole treated sample E2, it is clear that there is a cluster of bright bands in the first half of the movie (Figure 5E) that are removed by the standard deviation filter (Figure 5F). Examining the averages in the graph below (Figure 5G), the ratio of flow in the unfiltered data is 0.034 ± 0.05 and after filtering it becomes 0.01 ± 0.006. Thus, filtering reduces the standard deviation of this sample by 10-fold and brings the average for this sample to a value that is in line with the rest of the nocodazole treated samples (Figure 5G). This shows that filtering the data has a minimal impact on the overall estimates of motion, but decreases spurious results that may appear as false positives in a large-scale screen.

**Figure 6:**
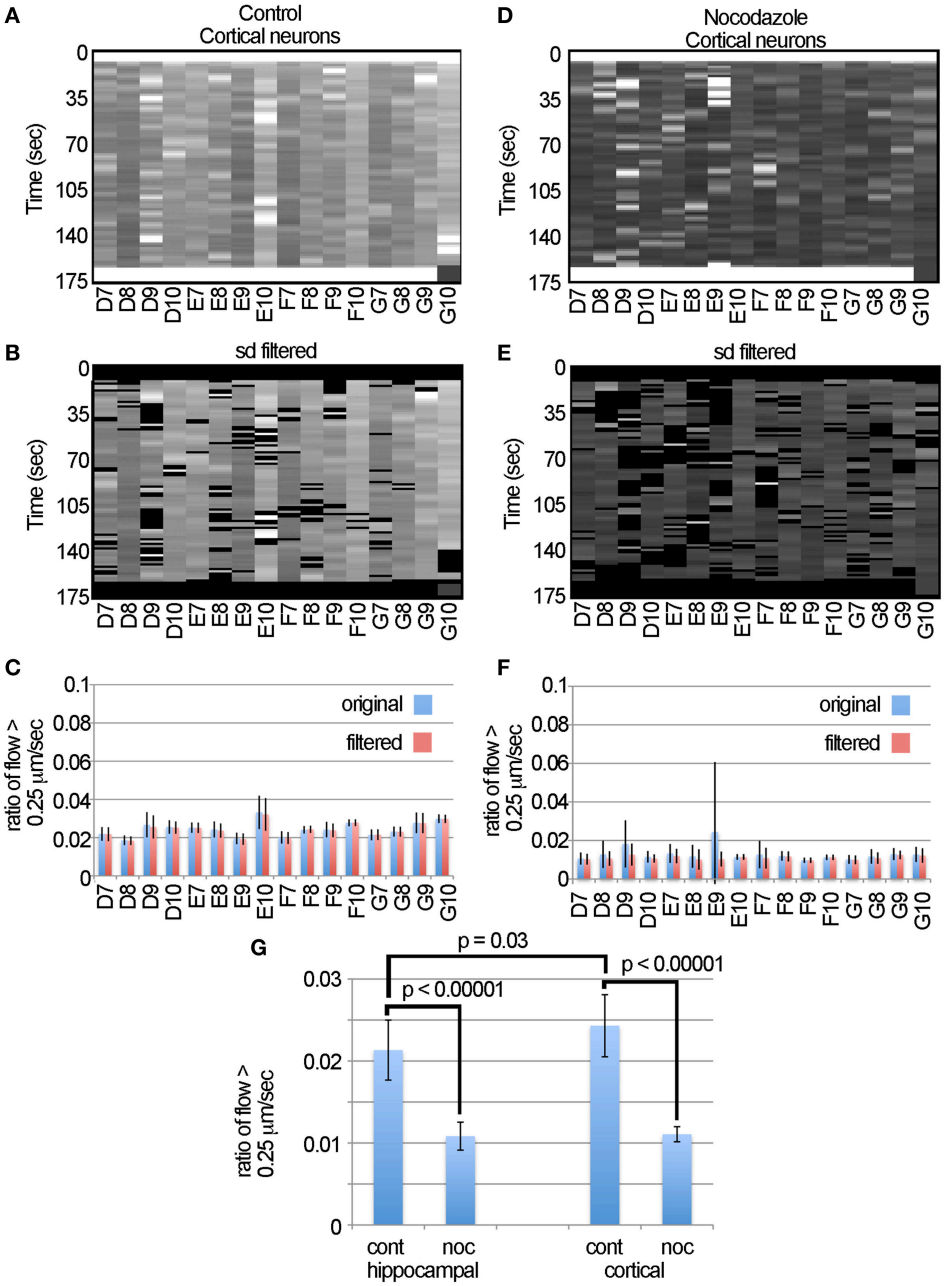
**Nocodazole causes similar reductions in transport in cortical and hippocampal neurons. (A)** Unfiltered flow table for control cortical neurons. **(B)** Filtered flow table for control cortical neurons. **(C)** Average flows for each sample, based on the unfiltered and filtered analysis of the control cortical neurons. **(D)** Unfiltered flow table for nocodazole treated cortical neurons. **(E)** Filtered flow table for nocodazole treated cortical neurons. **(F)** Average flows for each nocodazole treated sample. **(G)** A comparison of fast mitochondrial transport between cortical and hippocampal neurons and the effect on each of disrupting transport with nocodazole based on the ratio of flow > 0.25 μm/s.

To test if automated approaches for measuring motion can be used for different types of neurons, we then examined transport in cortical neurons. The data (Figures 6A,B,D,E) suggest that this algorithm consistently detected the decreased ratio of flow > 0.25 μm/s induced by nocodazole in cortical neurons as is seen in the hippocampal neurons. Likewise, examination of the flow tables (Figures 6D,E) and graphs (Figures 6C,F) indicates that filtering the data was effective in removing the outliers, particularly in sample E9, but had little effect on the overall profile of the data. To compare fast transport between hippocampal and cortical neurons and the effects of nocodazole on each, we plotted the average ratio of flow > 0.25 μm/s (Figure 6G). We found the cortical neurons had a slightly higher level of fast transport (0.024 ± 0.01, ave ± sd, *n* = 16) than the hippocampal neurons (0.021 ± 0.01, ave ± sd, *n* = 16) and that this difference was significant based on a two-tailed Student's *t*-test (*p* = 0.03). Likewise, nocodazole significantly (*p* < 0.00001) decreased the ratio of flow > 0.25 μm/s in both samples by approximately 60%. Altogether, this global analysis suggests that the use of the LKMTA is a useful approach for detecting the expected reduction in fast axonal transport induced by nocodazole in different types of neurons and that using the standard deviation filter is effective in removing time-lapse imaging artifacts that arise when using a robotic microscope.

### Large-scale statistical analysis by dunnett's test indicates nocodazole significantly reduced fast transport in all samples

**Figure 7:**
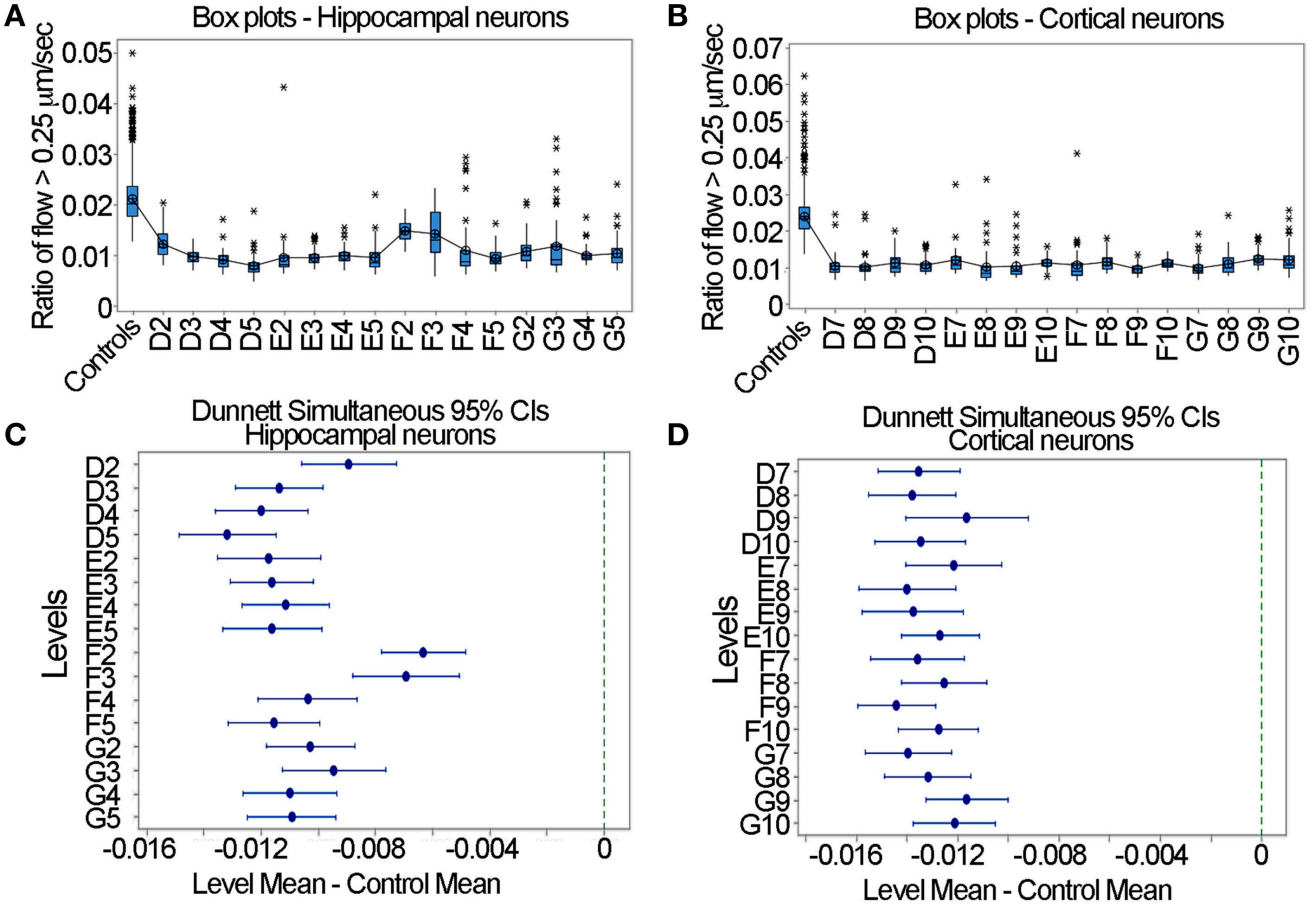
**Dunnett's test is useful for identifying reduced transport in large data sets. (A)** Box plots for the hippocampal neurons. **(B)** Box plots for the cortical neurons. **(C)** Dunnett's test for hippocampal neurons. **(D)** Dunnett's test for cortical neurons. The lack of overlap between the samples and the dashed lines in **(C,D)** indicates a significant reduction in transport.

In the context of the designing of a large-scale screen where one is seeking to determine whether a drug increases or decreases mitochondrial motility, the question arises as to acceptable formats of experimental design to detect effects. A standard and robust method for determining whether a given treatment has a significant effect relative to control samples is Dunnett's test (Dunnett, 1964). In this test, one pools the control data, determines the mean and variance, calculates the means and 95% confidence intervals for each of the experimental samples and determines if these overlap with the control. It is to be noted that this test is appropriate when the sole interest is in comparing each of the experimental groups to a single control group (Wallenstein et al., 1980). In Figures 7A,B the box plots of the ratio of flow > 0.25 μm/s are shown for the pooled controls and each nocodazole treated sample. For both the hippocampal and cortical neurons, the ratio was at approximately 0.02 for the controls and decreased to roughly 0.01 after nocodazole treatment. Using Dunnett's test with a One-Way ANOVA in Minitab, we found that in each case, nocodazole significantly decreased fast mitochondrial transport relative to the control samples in both the hippocampal and cortical neurons. This is evident by the lack of overlap between the error bars for each sample and the dashed line on the right hand side of the graph at a value of 0 (Figures 7C,D). The consistency of these results suggests that the output generated by the LKMTA can be reliably used to assess changes in the overall level of fast mitochondrial transport in a large-scale screen.

### The application of the difference tracker plugin to a large data set

**Figure 8:**
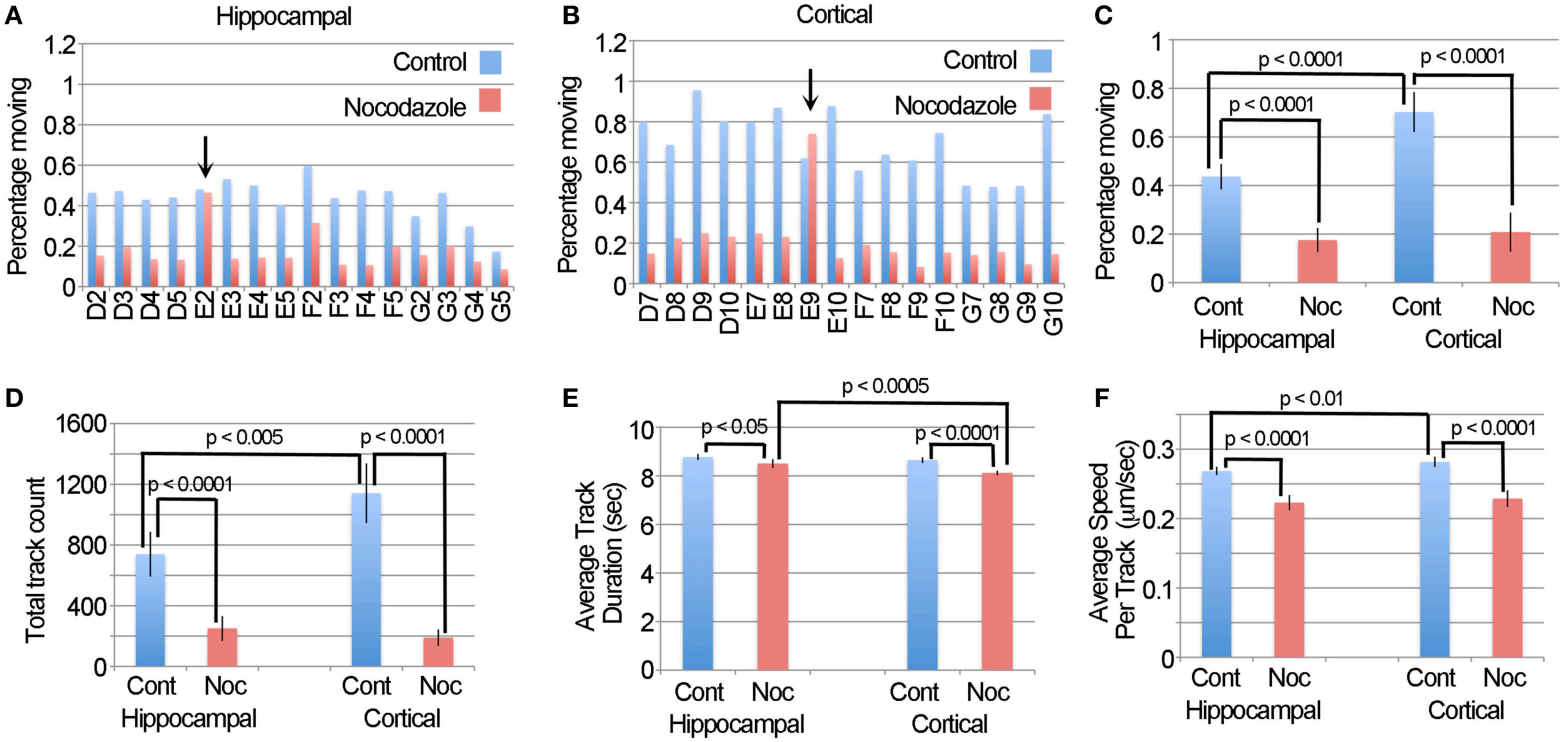
**The DTA generates results that are similar to the LKMTA. (A)** The percentage of moving mitochondria in hippocampal neurons before and after treatment with nocodazole as assessed by the DTA. The arrow points out the same well, as above, where spurious transport was observed. **(B)** The same data, but for cortical neurons. **(C)** A comparison of the percentage of moving mitochondria as function of cell type and nocodazole treatment. **(D)** Average track counts. **(E)** Average track durations. **(F)** Average speed per track. All error bars are 95% ci.

A challenge in validating the LKMTA as a tool for assessing large data sets is that it is not practical to use manual tracking on large data sets. We reasoned that an alternative approach would be to compare the output of the DTA with the output of the LKMTA on the same data set described above. If both gave similar results, it would provide confidence that both are reliable. Likewise, large variations in the output between the approaches would call one or both into question. To make this comparison, we took the movies that had been pre-processed for the LKMTA (i.e., binned by a factor of four in x and y, registered with StackReg, cropped and stretched on the time axis by a factor of two) as our starting point for analysis by the DTA. Based on guidelines on how to process images using the DTA (Andrews et al., 2010; Bros et al., 2015), we first ran the Enhance Contrast plugin using the settings of saturated pixels 5% and equalize histogram on all of the images and then converted them to a bit depth of 8. We then ran the Difference Filter with the settings Minimum difference 20 and Difference frame offset 4 on the movies. As mentioned before, this subtracts out mitochondria that do not move over a distance of at least 1 pixel over a period of 4 frames (i.e., 0.432 μm/7 s or 0.06 μm/s). The plugin then thresholds the image and produces a table that contains detailed information about the stationary (i.e., static population) and the moving population of mitochondria. This provides an estimate of the percentage of moving mitochondria that is analogous to the output of the LKMTA. In addition, we note that the processing time for the DTA plugins was also at a rate of about 1 s per frame. Starting with the individual wells for the hippocampal and cortical neurons (Figures 8A,B), we observed that nocodazole produced a fairly consistent reduction in the percentage of moving mitochondria; the two exceptions, E2 and E9, were the same wells where the LKMTA produced spurious output before the data was filtered. Taking the averages for the datasets and plotting them in Figure 8C, the percentage of moving mitochondria in the hippocampal neurons before the addition of nocodazole was 0.43 ± 0.05% (ave ± 95% ci), after treatment, this was reduced to 0.17 ± 0.05%; a 60% reduction. Likewise for the cortical neurons the percentages were 0.70 ± 0.08 and 0.21 ± 0.08%, which was a 71% reduction. In comparing this to the unfiltered output of the LKMTA, the percentage of moving mitochondria in the hippocampal neurons before the addition of nocodazole was 2.1 ± 0.2% (ave ± 95% ci), after treatment this was reduced to 1.3 ± 0.3%; a 41% reduction. Likewise for the cortical neurons the percentages were 2.5 ± 0.2 and 1.3 ± 0.2%, which was a 48% reduction. Overall, the reduction in transport induced by nocodazole as measured by the LKMTA and the DTA were similar (i.e., 45% vs. 65% respectively). The primary difference was that the absolute estimated levels of transport using the LKMTA were approximately 5 times higher than the estimates using the DTA. We note that this was expected as the DTA has more stringent requirements than the LKMTA for classifying a mitochondrion as moving by fast transport. The major advantage the DTA has over the LKMTA is that in addition to producing information about the percentage of mitochondria in motion, it generates output pertaining to the number of tracks followed, the average duration of the tracks and their velocity. Similar to the output of the percentage of moving mitochondria, the DTA reported significant reductions in the total track count following treatment with nocodazole and a higher total track count in cortical neurons (Figure 8D). While there were also effects on track duration as the result of nocodazole, they were very small (Figure 8E). Likewise, while there were reductions in average track speeds they were modest (Figure 8F). As Bros et al. (2015) has suggested that DTA may be underestimating changes in track length and velocity, it seems reasonable that these significant effects may be larger in absolute terms. Altogether, this confirms that automated approaches are relatively weak in estimating of the absolute values for fast transport, but strong in detecting relative changes (Bros et al., 2015).

## Discussion

The purpose of this study was to develop methodologies for the analysis of fast mitochondrial transport from data acquired using a robotic microscope (Mei et al., 2012). We addressed questions of measurement precision, speed, reliably, workflow ease, statistical processing and presentation. In considering different approaches, we chose to measure optical flow with the classic Lucas Kanade algorithm in the FlowJ (Abràmoff et al., 2000) and single particle tracking with the Difference Tracker plugins (Andrews et al., 2010). We did so because others had demonstrated that they are capable of tracking mitochondrial motion (Gerencser and Nicholls, 2008; Andrews et al., 2010; Bros et al., 2015), they are implemented as free open source plugins in ImageJ and they both had the potential to rapidly generate complete, but concise descriptions of mitochondrial movement that required minimal user supervision. Using these, we confirmed that both are capable of detecting the motion of a single mitochondrion (Figures 1, 2), the effect of disrupting fast mitochondrial transport by nocodazole (Figures 3, 4) and could consistently detect the disruption of fast transport by nocodazole in a large-scale screen (Figures 5–8). In the course of testing these algorithms, we additionally developed methods for filtering the data to remove spurious results that arose from changes in focus, stage position, and illumination that are, to the best of our knowledge, novel. This work validates that fast mitochondrial transport in neurons can be rapidly assessed in an automated manner using data acquired with a robotic microscope, which provides a proof of concept demonstration for future large-scale screens of compounds that modulate mitochondrial motility.

In developing an automated process for measuring fast mitochondrial transport the foremost concern is that the output is reliable. To address this we first examined the motion of a mitochondrion using the LKMTA, the DTA, by kymograph and manual point selection (Figure 2). We found that all four approaches gave similar, but not identical, estimates for its motion. This confirms fast transport is measurable by optical flow and particle tracking (Gerencser and Nicholls, 2008; Lihavainen et al., 2012), but raises the caution that the absolute values of motion estimates depend on the specific approach. In comparing these approaches a natural question is, “which is the best for analyzing mitochondrial motion?” As of now, the strengths of the LKMTA are that it analyzes motion over a relatively large velocity range and using it we have developed procedures to filter out spurious data. The strength of the DTA is that it provides a more focused analysis of rapid motion and additional information about transport such as run duration. The advantage of both over manual tracking is that it they are less labor intensive and prone to unconscious bias, nonetheless at this time manual tracking seems to provide the most accurate estimates of mitochondrial transport. Thus, instead of asking which approach is best, it may be better to utilize a variety of approaches on the same data set and use the results from each approach in accordance to their strengths.

In addition to combining data from various approaches to better describe mitochondrial transport, directly improving the approaches themselves would also be straightforward and helpful for future studies. In particular, while our experiments indicate that the LKMTA is useful for motion tracking it is by no means state of the art. In this regard, one aspect of the optical flow community that is admirable is that as various approaches are developed they are tested in terms of speed and reliability against a set of standard movies (Baker et al., 2011). In particular, the Middlebury Optical Flow website (http://vision.middlebury.edu/flow/) currently has a list comparing 106 algorithms. Sorting the list to identify fast and precise algorithms suggests that the Component Fusion, the EPPM w/o HM (i.e., Fast Edge-Preserving PatchMatch for Large Displacement Optical Flow) (Bao et al., 2014) and SimpleFlow (Tao et al., 2012) would be particularly useful to consider. They have fast processing times, less than 10 s, while having a precision that is only slightly inferior to algorithms, such as the NNF-Local, that has a processing time of 10 min for a single pair of images. We note that for our analysis the processing time for motion tracking was approximately 1 frame per second and that the processing of 64 movies took about 1 h for both the LKMTA and DTA. If we were to utilize an approach that required 10 min per frame, it would take 25 days to process the same set of data. Thus, a balance between processing time and accuracy must be considered. Likewise, given the vigor with which particle tracking routines are being improved (LIRIS)^1^ it seems reasonable that there may be better software that could be applied to this problem. In these terms, building on what has been done with the DTA and LKMTA in ImageJ/FIJI would be particularly useful as it would increase the accessibly of robust tracking tools for biologists.

While precision is important, there are common problems associated with time-lapse microscopy that make the automated analysis of motion difficult. In particular, stage drift, minor transient changes in the intensity of illumination and changes in focus all produce spurious output that appears as robust optical flow. Since a goal of the automated transport analysis is to discover compounds that alter mitochondrial transport, procedures need to be in place to automatically deal with these problems to minimize the occurrence of false positives. Accordingly, as a pre-processing step we used StackReg to minimize stage drift (Thévenaz et al., 1998). Nonetheless, even after image registration, small global shifts in the images remained, as well as the problems of focus and flicker. While one could apply a median filter to the final data in the flow tables (e.g., Figures 4B–E), we thought it would be interesting to attempt a more sophisticated approach based on the standard deviation of the velocity histograms over time (Figure 4). For most of the samples, this had a negligible effect on the measured flow (Figure 5); nonetheless this approach was effective in filtering out large changes in the velocity profiles that created large standard deviations for particular samples (e.g., sample E2 in Figure 5E). While this approach could be refined, we think the inclusion of approaches to filter out spurious data will be needed for decreasing the incidence of false positives in future large-scale screens.

As a means to evaluate the reliability of the algorithms in a screen, we then asked the question of whether the DTA and LKMTA were capable of consistently detecting the disruption of fast axonal transport induced by nocodazole (Morris and Hollenbeck, 1995; Ligon and Steward, 2000). While there were quantitative differences in the output, the overall similarity of two independent automated means of assessing transport increases one's confidence that they both are capable of measuring mitochondrial transport in an automated fashion. In terms of conducting a large-scale screen, either or both approaches appear to provide a means to identify compounds or mutations that are of interest. In turn, it seems reasonable that once identified, at this point in time, a more careful manual analysis would need to follow to more completely characterize the effects of novel compounds on transport.

Exciting advances have been made in our understanding of mitochondrial transport over the last decade, nonetheless one challenge in this field is that acquiring and processing the data needed to study this topic is labor intensive. Combining robotic acquisition of data with unbiased automated analysis has the promise to transform the scale of future studies and usher in an era of “transport-omics” that we believe will facilitate drug discoveries for neurodegenerative diseases such as Alzheimer's, Parkinson's and ALS.

## Author contributions

SP and KM designed the project, XL and SP conducted experiments, KM analyzed the data and KM and SP wrote the paper.

### Conflict of interest statement

The authors declare that the research was conducted in the absence of any commercial or financial relationships that could be construed as a potential conflict of interest.

## Footnotes

^1^Liris, F. The Visual Object Tracking VOT2014 Challenge Results.
