# Supplementary material for: Automated measurement of fast mitochondrial transport in neurons
Source: Front Cell Neurosci. 2015 Nov 3;9:435. doi: 10.3389/fncel.2015.00435 (PMC4630299; doi:10.3389/fncel.2015.00435)
Supplement: Supplementary file 3 [file Presentation1.PDF]

Supplemental Information
Miller et al

Supplementary Figure

Supplemental Discussion

Supplementary movies

Supplemental References

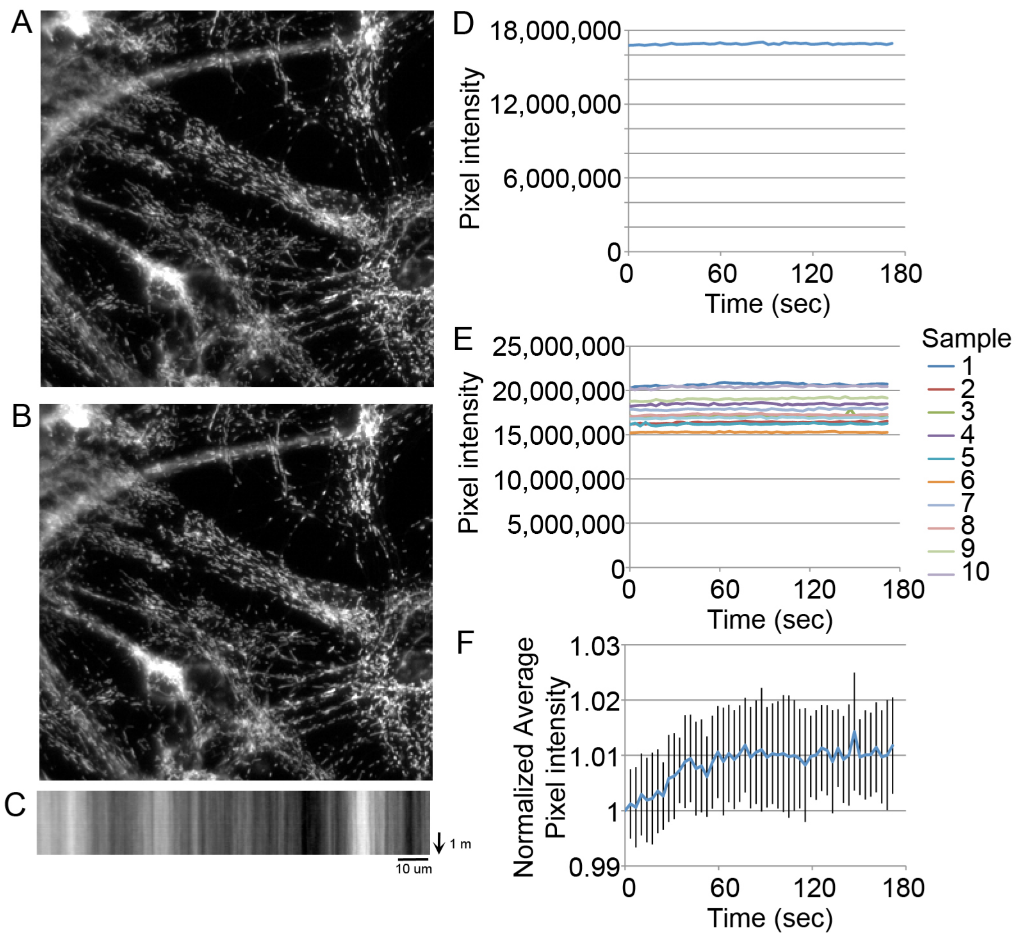

Supplemental Figure 1. Negligible photo-bleaching is occurring in our study. A. The first frame from a time-lapse movie. B. The last frame of the movie. C. A Summed Z-projection kymograph of the movie; arrow = 1 min; bar = 10 μm. D. The total sum of the pixel intensity over time for the example shown in panels A-C. E. The total sum of pixel intensity over time for 10 samples. F. The normalized average +/- standard deviation of the pixel intensity for the samples.

Supplemental Discussion

A key assumption in the underlying mathematics used for the LKMTA is that brightness of the image does not change between time steps (Gennert and Negahdaripour, 1987). Nonetheless, because fluorescent time-lapse studies illuminate samples with bright light, photo-bleaching is a common problem (Hoebe et al., 2007). In addition to negatively affecting the tracking algorithm this could also lead to photo-damage that jeopardizes the health of neurons. To test if photo-bleaching occurred in our studies, we examined how the total sum of pixel intensity changed over time. In Supplementary Figure 1 the first and last images in a time-lapse series are shown (Sup.

Fig. 1A and 1B). As a first means to test for signs of photo-bleaching, we made a Z-projection of the entire movie to produce a kymograph (Supplementary Figure 1C). The lack of change in pixel intensity as a function of time suggested that if photo-bleaching occurred it was subtle. To examine this quantitatively, we then calculated the sum of pixel intensity for each time point in the movie (Supplementary Figure 1D). The graph indicates that photo-bleaching was negligible. To test this more systematically, the analysis was repeated in 10 samples (Supplementary Figure 1E). While the total pixel intensity differed from movie to movie, no observable decline was found in any of the samples. To examine how this data set behaved collectively, we normalized pixel intensity and calculated the average and standard deviation for the group n = 10 (Supplementary Figure 1F). Noting the scale ranged from 0.99 to 1.03, on average there was about a 1% increase in pixel intensity over the course of the experiment. This indicates that if photo-bleaching is occurring in our study it is below detectable limits and confirms findings from previous studies that have suggested the photo-bleaching is negligible with MitoTracker Green using typical imaging conditions (Pendergrass et al., 2004).

Supplementary movie 1. A movie of a moving mitochondrion. The time interval between frames is 1.75 secs and 1 pixel equals 0.432 microns.

Supplementary movie 2. A movie where the moving mitochondrion has been digitally removed in Photoshop.

Supplemental References

Gennert, M.A., and Negahdaripour, S. (1987). Relaxing the brightness constancy assumption in computing optical flow.

Hoebe, R., Van Oven, C., Gadella, T.W., Dhonukshe, P., Van Noorden, C., and Manders, E. (2007). Controlled light-exposure microscopy reduces photobleaching and phototoxicity in fluorescence live-cell imaging. *Nature biotechnology* 25**,** 249-253.

Pendergrass, W., Wolf, N., and Poot, M. (2004). Efficacy of MitoTracker Green™ and CMXRosamine to measure changes in mitochondrial membrane potentials in living cells and tissues. *Cytometry Part A* 61**,** 162-169.
